# From chaos to order: cross-kingdom coordination buffers microbial communities against typhoon impacts

**DOI:** 10.1186/s12866-026-05387-9

**Published:** 2026-07-11

**Authors:** Hui Huang, Junze Wu, Zhangxi Hu, Huaming Wu, Yulei Zhang

**Affiliations:** 1 College of Fisheries, Guangdong Ocean University, Zhanjiang, 524088 Guangdong China; 2 Guangdong Provincial Key Laboratory of Aquatic Animal Disease Control and Healthy Culture, Key Laboratory of Marine Ecology and Aquaculture Environment of Zhanjiang, Zhanjiang, 524088 China

**Keywords:** Typhoon disturbance, Microbial community dynamics, Ecosystem resilience, Cross-kingdom interactions

## Abstract

**Background:**

Typhoon disturbances profoundly reshape coastal microbial ecosystems; however, the coupled responses and co-successional dynamics of bacterial and phytoplankton communities remain poorly understood. Identifying their interactions and key drivers is essential for understanding ecosystem resilience and predicting post-disturbance algal dynamics.

**Methods:**

Using Zhanjiang Bay as a model system, we analyzed microbial succession across two typhoon events with contrasting intensities. High-throughput sequencing and environmental data were integrated with multiple machine learning models to identify the influence patterns of bacterial communities on phytoplankton diversity. SHAP analysis was applied to quantify feature contributions and detect threshold effects, and network analysis was used to characterize cross-kingdom interactions across successional stages.

**Results:**

Microbial communities exhibited consistent directional succession under both typhoons, although Prapiroon induced stronger displacement (2.38-fold in bacteria and 2.53-fold in phytoplankton). Random Forest achieved the best predictive performance (R^2^ = 0.74, RMSE = 0.23). Temperature and the rare taxon Bdellovibrionota were identified as key drivers, showing clear threshold effects: phytoplankton diversity declined above 30.76 °C and increased when Bdellovibrionota exceeded 0.02%. Diversity peaked immediately after disturbance and declined during recovery, indicating a stage-dependent regulatory shift. Microbial networks followed a "collapse–reorganization–recovery" trajectory, reflecting ecosystem resilience.

**Conclusion:**

Our findings reveal a predictable, stage-dependent framework of microbial succession under typhoon disturbance, driven by environmental filtering and biotic interactions. The identified thresholds and microbial indicators provide a basis for recognizing critical temporal windows of elevated bloom risk, highlighting the potential of integrating microbial data with machine learning for adaptive coastal management.

**Supplementary Information:**

The online version contains supplementary material available at https://doi.org/10.1186/s12866-026-05387-9.

## Background

Tropical cyclones (commonly termed typhoons or hurricanes) are among the most destructive natural disturbances to coastal and open-ocean ecosystems. These extreme events are characterized by intense winds, heavy precipitation, storm surges, induce drastic changes in hydrodynamic conditions that impose acute physical perturbations and biogeochemical stress on aquatic environments [1, 2]. During a typhoon, enhanced surface runoff and sediment resuspension rapidly alter water chemistry and light availability [3]. Simultaneously, the disruption of vertical stratification reshapes the distribution and transport of plankton. Such direct and indirect effects profoundly modify the composition, structure, and functional attributes of prokaryotic and eukaryotic organisms [4]. Given their role as foundational drivers of biogeochemical cycling and primary production, elucidating microbial responses to typhoon disturbances is critical for predicting the resilience of coastal ecosystems under an increasingly unpredictable climate [5, 6].

While typhoons are recognized as major drivers of local microbial dynamics, many studies have focused on single microbial groups or abiotic factors in isolation. For example, typhoon-induced harmful algal blooms are frequently reported and often attributed to nutrient pulses from terrestrial runoff and upwelling [7, 8]. Similarly, post-typhoon shifts in bacterial community composition have been linked to changes in organic matter availability and salinity [9]. Beyond taxonomic shifts, whether microbial communities respond to typhoon disturbances through deterministic or stochastic processes remains an open question. Some studies attribute post-disturbance changes to enhanced environmental filtering, resulting in consistent successional patterns [10], whereas others emphasize that community reassembly following disturbance may be shaped by initial conditions and stochastic dispersal, leading to substantial divergence in community structure [11]. However, these separate lines of inquiry rarely address the coupled dynamics and potential nonlinear interactions among bacteria, phytoplankton, and environmental factors. Bacteria–phytoplankton interactions (including competition, predation, and mutualism) are central to nutrient regeneration, organic matter turnover, and community stability [12]. Filling this critical knowledge gap requires temporally resolved observations across multiple typhoon events, coupled with species-level tracking and analytical frameworks capable of disentangling these high-dimensional ecological interactions.

This study focuses on Zhanjiang Bay, a representative coastal region in the northwestern South China Sea that is frequently impacted by tropical cyclones—averaging up to 10 direct or indirect typhoon influences annually [13]. Previous studies have characterized the microbial responses associated with Typhoon Prapiroon in this region [14, 15]. Building upon these earlier findings, we conducted a comparative, longitudinal investigation of two independent typhoon events—Typhoon Prapiroon (July 2024) and Typhoon Wipha (July 2025)—to systematically resolve the dynamic responses of bacterioplankton and eukaryotic phytoplankton communities and assess the reproducibility of disturbance-driven ecological patterns. By integrating metabarcoding sequencing, environmental monitoring, and artificial intelligence–based analytical approaches, we comprehensively characterized microbial and phytoplankton community dynamics across spatial and temporal scales. While AI-based frameworks have been applied to uncover complex ecological associations within large-scale datasets [16, 17], their potential for evaluating ecosystem resilience under extreme disturbance events remains largely unexplored. Specifically, this work aimed to: (i) compare microbial community response patterns under different typhoon disturbances,(ii) identify key biotic and abiotic drivers shaping community interactions and quantify their stage-specific contributions,and (iii) reconstruct the successional trajectories of bacteria–environment–phytoplankton interactions following disturbance. Our results provide new insights into the ecological mechanisms underlying ecosystem stability and offer valuable implications for predicting and managing coastal ecosystems under future climate-change scenarios.

## Methods

### Study area and sampling

**Fig. 1:**
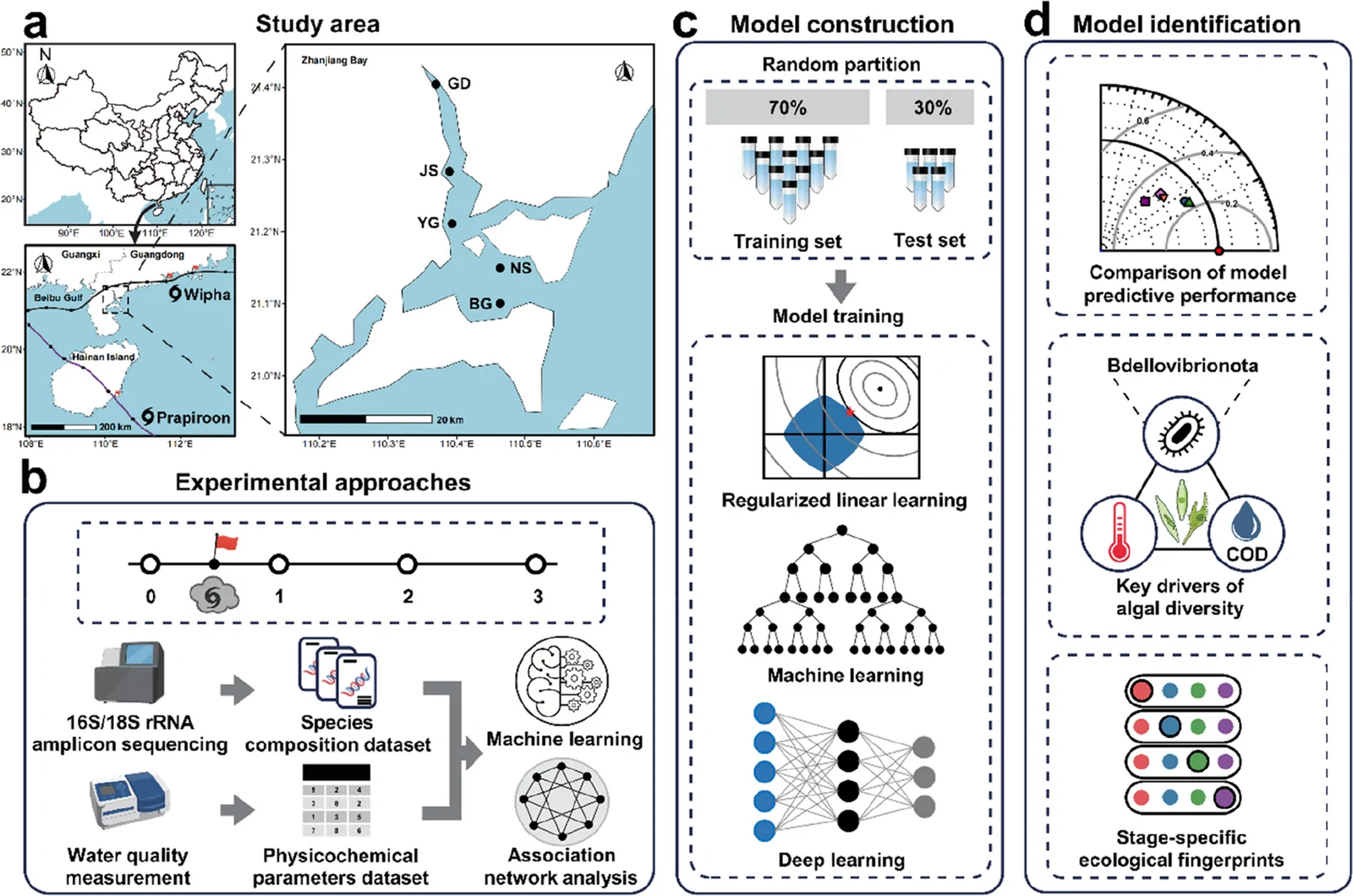
Schematic overview of the study design, model selection, and model-based ecological interpretation **a** Typhoon track and sampling station locations in Zhanjiang Bay, Guangdong Province, China. Red flags indicate typhoon landfall locations. The purple and black lines represent the tracks of Typhoon Prapiroon and Typhoon Wipha, respectively. **b** An analytical workflow integrating 16S/18S rRNA amplicon sequencing and water quality measurements for machine learning-based prediction of phytoplankton diversity dynamics and evaluation of association networks. **c** Datasets were randomly split into training (70%) and testing (30%) sets for model training using regularized linear models, machine learning algorithms, and deep learning architectures. **d** Comparison of multi-model predictive performance and ecological interpretation, followed by model-based identification of key drivers and stage-specific ecological fingerprints (Bdellovibrionota and Temperature). Key ecological chain linking Bdellovibrionota, Temperature, COD, and algal. Parts of panels b and c were generated using BioGDP (https://biogdp.com)

Four sampling stages were defined for each typhoon event: Stage 0 (2–3 days prior to landfall), Stage 1 (3 days post-landfall), Stage 2 (10 days post-landfall), and Stage 3 (15–16 days post-landfall). Field campaigns for Prapiroon were conducted on July 20, July 25, August 1, and August 7, 2024, whereas those for Wipha occurred on July 17, July 23, July 30, and August 4, 2025. At their respective first landfall points, maximum sustained wind speeds reached 28 m·s⁻^1^ (Prapiroon) and 30 m·s⁻^1^ (Wipha). Typhoon track and intensity data were obtained from the China Meteorological Administration (https://typhoon.weather.com.cn/gis/typhoon_m.shtml). These events occurred in comparable seasonal periods approximately one year apart, but followed distinct trajectories along opposite sides of the bay; identical sampling intervals relative to landfall enabled direct comparisons of successional dynamics under similar environmental contexts (Fig. 1a–b).

For each typhoon, triplicate surface (S, 0.5 m) and bottom (B, approximately 1 m above the seafloor) water samples were collected from five stations spanning Zhanjiang Bay (from north to south) using an acrylic water sampler (Qingdao Loo-bo Environmental Protection Group Co., Ltd., China). Each individual sample was obtained by filtering 500 mL of seawater through 0.22 μm polycarbonate membranes (Pall Corporation, USA). The filters were immediately flash-frozen and stored at −80 °C for subsequent analysis. This resulted in 30 samples per stage and 120 samples per event (240 in total).

### Environmental characterization and microbial community profiling

Physicochemical parameters, including temperature, salinity, dissolved oxygen (DO), pH, and water depth, were measured in situ using portable analyzers (LH-T600, Zhejiang Lohand Environment Technology Co., Ltd., Hangzhou, China) and a depth sounder (SM-5A, Speedtech, St. George, UT, USA) (Fig. 1b). In the laboratory, concentrations of chlorophyll a (Chl a), chemical oxygen demand (COD), and dissolved nutrients—including silicate (SiO₃^2^⁻), phosphate (PO₄^3^⁻), nitrate (NO₃⁻), nitrite (NO₂⁻), and ammonium (NH₄⁺) were determined according to National Standard Methods [18].

Total genomic DNA was extracted using the E.Z.N.A.® Water DNA Kit (Omega Bio-tek, USA). To characterize microbial communities, the V3–V4 region of the bacterial 16S rRNA gene (primers 341F: 5'-CCTACGGGNGGCWGCAG-3' and 806R: 5'-GGACTACHVGGGTATCTAAT-3') and the V9 region of the eukaryotic 18S rRNA gene (primers V9F: 5'-CCCTGCCNTTTGTACACAC-3' and V9R: 5'-CCTTCNGCAGGTTCACCTAC-3') were amplified in triplicate. Briefly, each 20 μL PCR mixture contained 4 μL of 5× FastPfu Buffer, 2 μL of 2.5 mM dNTPs, 0.8 μL of each primer (5 μM), 0.4 μL of FastPfu Polymerase, and 10 ng of template DNA. Cycling conditions consisted of an initial denaturation at 95 °C for 2 min, followed by 25 cycles of 95 °C for 30 s, 55 °C for 30 s, and 72 °C for 30 s, with a final extension at 72 °C for 5 min. Amplicons were purified using the AxyPrep DNA Gel Extraction Kit (Axygen Biosciences, USA) and quantified using a Qubit 3.0 fluorometer (Invitrogen, USA). Triplicate PCR products were pooled prior to indexing PCR. The indexed PCR products were then purified, and equimolar amounts of the purified amplicons were used for library preparation. Sequencing (2 × 300 bp) was performed on an Illumina platform (Shanghai Biozeron, China).

Raw sequences were demultiplexed using Trimmomatic and in-house Perl scripts [19]. Quality control criteria included a 10-bp sliding window (quality score < 20) and a minimum length of 50 bp, followed by exact barcode matching. Processed reads were analyzed via the DADA2 algorithm within the QIIME 2 (2020.11) framework (maxEE = 2) to identify amplicon sequence variants (ASVs) [20, 21]. Taxonomic assignment was conducted using the uclust algorithm against the Silva (SSU138.1 for 16S,SSU138.2 for 18S) database with an 80% confidence threshold [22, 23, 24]. Phytoplankton ASVs were specifically binned and normalized based on the minimum library size to facilitate cross-sample comparisons. Functional profiles were predicted using PICRUSt2 (v2.6.3) based on the KEGG database [25].

### Statistical analysis and machine learning frameworks

#### Microbial community analysis

All data analyses and result presentations were performed using R (v 4.5.0). To minimize noise from low-abundance taxa, only ASVs with >0.1% relative abundance in at least 20% of samples—appearing in at least two samples per typhoon event—were retained. The Wilcoxon rank-sum test was then used to perform sample-level comparisons of relative abundance differences between the two typhoons (based on raw values), and p-values were adjusted for multiple testing using the Benjamini-Hochberg (BH) method [26]. Effect sizes were quantified as the log2 fold change (log2FC) of Wipha relative to Prapiroon, with 95% confidence intervals derived from the 2.5th and 97.5th percentiles of 1,000 bootstrap resamplings [27]. Variation in alpha diversity across stages was evaluated using the Kruskal-Wallis test, followed by Dunn’s post-hoc multiple comparisons with FDR correction. To quantify community structural divergence, permutational multivariate analysis of variance (PERMANOVA) was performed with 999 permutations [28], visualized via non-metric multidimensional scaling (NMDS, k = 2) based on Bray-Curtis dissimilarities. Furthermore, to evaluate the congruence of community trajectories between typhoons, Principal Coordinates Analysis (PCoA) was conducted on separate Bray-Curtis distance matrices. Procrustes analysis was then employed to optimally align these PCoA spatial structures, assessing the consistency of successional directions and change patterns. The Procrustes correlation (r), similarity index (R^2^), and statistical significance were rigorously evaluated through 999 permutations [29].

#### Modelling and evaluating the coupled dynamics of Bacterial and Phytoplankton communities

To characterize ecological relationships within microbial communities, we benchmarked multiple machine learning architectures (Fig. 1c). These models were used to capture key ecological associations from high-dimensional bacterial and environmental datasets to predict phytoplankton diversity dynamics [30]. All input features, including bacterial taxon-level relative abundances and environmental variables, were standardized prior to analysis, and the dataset was split into training and testing sets (7:3 ratio). To ensure robust performance, hyperparameter optimization and evaluation were conducted via 5-fold cross-validation [31]. For the Prapiroon dataset, ElasticNet optimized α (0–1, step 0.05) and λ (50 log-scale candidates) through grid search based on Mean Absolute Error (MAE). Random Forest (RF) hyperparameter space included mtry, ntree, nodesize, and maxnodes (900 combinations),utilizing the Residual Sum of Squares (RSS) for splitting and Root Mean Square Error (RMSE) for validation. XGBoost (eXtreme Gradient Boosting) optimization covered 1,944 combinations across eta, max_depth, nrounds, and subsample, using Mean Squared Error (MSE) loss and MAE for validation. Support Vector Machine (SVM) regression employed a radial basis function (RBF) kernel, with grid search optimizing the penalty parameter C and kernel width γ. A secondary optimization round was performed on the full-feature dataset (parameter details in Table S1).

For the deep learning component, we developed a prediction framework based on a fully connected Deep Neural Network (DNN) utilizing a hierarchical feedforward architecture, which comprises a shared feature extraction module and a task-specific prediction head [32]. Input features were initially projected into a 128-dimensional latent space, with ReLU activation and dropout regularization (0.3) applied to stabilize feature representation and mitigate overfitting. To capture higher-order nonlinear dependencies within bacteria-environment interactions, the network employed a progressively contracting hidden structure, ultimately mapping the 32-dimensional extracted features linearly to yield continuous predictions of phytoplankton Shannon diversity. The model was optimized using the Adam optimizer (learning rate = 0.001) minimizing mean squared error (MSE),to ensure robust generalization, an early stopping criterion (patience = 20) was implemented based on test set performance.

The SHAP (SHapley Additive exPlanations) framework, grounded in cooperative game theory, decomposes model predictions into additive contributions of individual input features by evaluating their marginal effects across all possible feature subsets [33] (Fig. 1d). This approach effectively captures nonlinear, high-dimensional interactions within complex models, providing a mechanistically interpretable link to elucidate post-disturbance ecological succession [34]. In this study, all machine learning architectures and SHAP interpretations were implemented using Python (v3.13.9) and PyTorch (v2.9.1).

#### Panoramic view of Bacteria-Phytoplankton association networks

To systematically resolve the ecological association structures among bacteria, phytoplankton, and environmental factors, we constructed ecological association networks for four distinct stages based on the full-feature dataset (Fig. 1b). Spearman correlation coefficients and their significance were calculated for all pairwise feature combinations, with multiple testing corrected using the BH method [35]. During network construction, bacteria, environmental factors, and phytoplankton were treated as three distinct node types. To enhance network readability and focus on biologically meaningful associations, only significant edges (FDR-corrected P < 0.05) and correlation strengths exceeding the upper 25th percentile of the global distribution of absolute correlation coefficients were retained [36]. To improve comparability among nodes, node size was scaled based on normalized connectivity.

## Results

### Microbial community structure under different typhoon events

All samples passed high-throughput sequencing quality control and were included in subsequent analyses (Section 2.2). In total, 159,215 bacterial ASVs and 363,260 phytoplankton ASVs were obtained, which were assigned to 42 bacterial phyla and 224 eukaryotic phytoplankton genera across eight phyla. Bacterial phyla unique to the Prapiroon event included Acetothermia, Caldisericota, Cloacimonadota, and Deferribacterota, whereas Modulibacteria was exclusively detected during the Wipha event (Fig. S1). No phytoplankton phyla were unique to either event; however, Wipha and Prapiroon harbored 52 and 45 unique genera, respectively, with 127 genera shared between the two events. Given that bacterial phylogenetic frameworks and taxonomic annotation often entail substantial uncertainty at finer taxonomic levels [37, 38], bacterial analyses were therefore conducted at the phylum level. In contrast, eukaryotic phytoplankton exhibit greater stability and interpretability at the genus level [39], and thus phytoplankton analyses were conducted at the genus level.

**Fig. 2:**
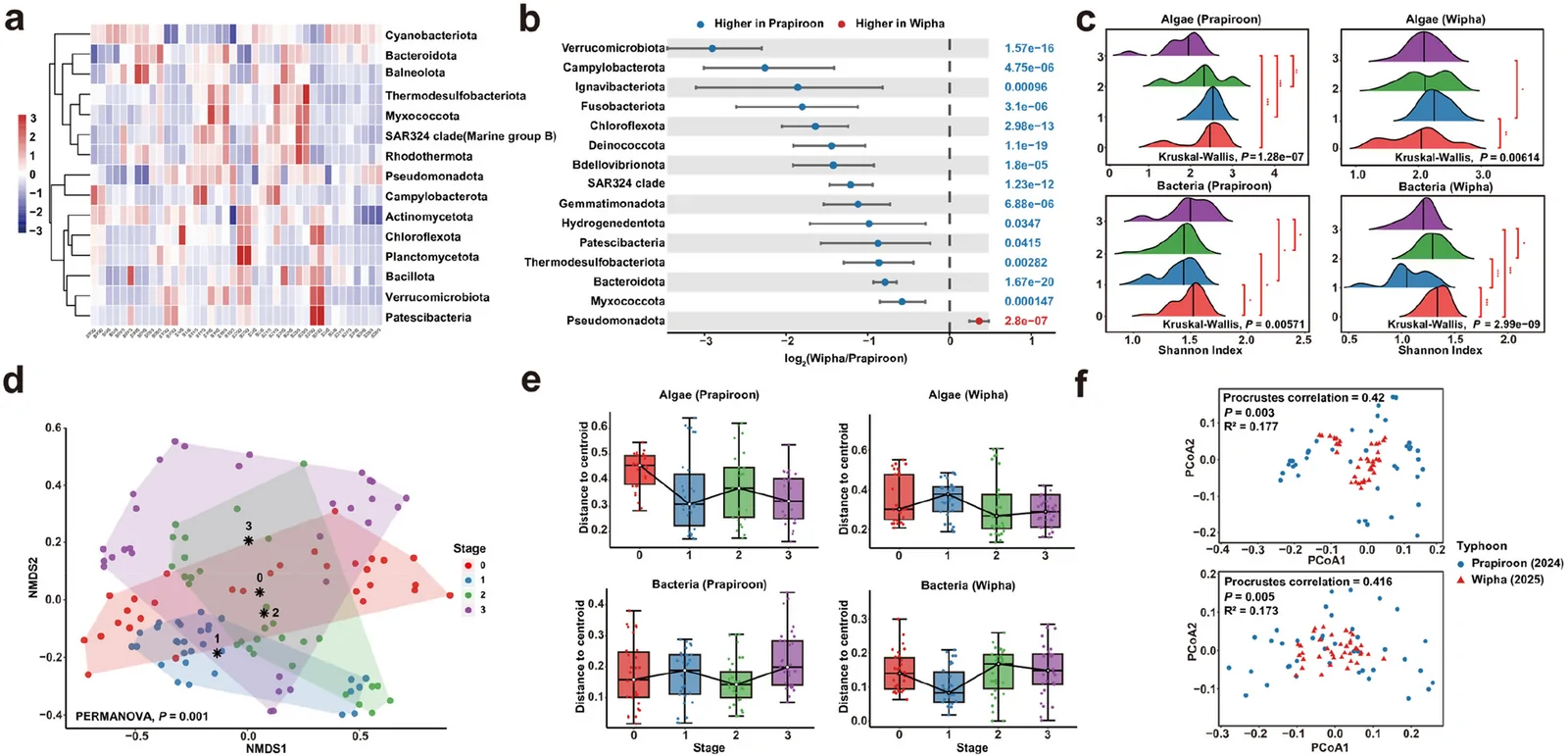
Comparison of species’ spatiotemporal distributions and response patterns under different typhoon events. **a** Mean relative abundance of the top 15 bacterial phyla during Typhoon Wipha (3 technical replicates). Values were Z-score standardized (range −3 to 3) to highlight compositional differences across samples. **b** Differential bacterial phyla between the two typhoons. Error bars represent 95% confidence intervals, and the dashed line indicates the no-difference baseline. Log_2_ effect sizes indicate the magnitude and direction of differences in relative abundance between Typhoon Wipha relative to Typhoon Prapiroon. Values on the right represent adjusted p-values from Wilcoxon rank-sum tests. **c** Kernel density distributions of Shannon diversity index. Vertical lines represent medians, and stages are distinguished by color. Asterisks denote significance levels of pairwise comparisons (* *P* < 0.05, ** *P* < 0.01, *** *P* < 0.001). **d** Distribution of algal communities during Typhoon Prapiroon. Convex hulls represent the community range for each stage, and centroids indicate the geometric center of each stage. **e** Within-group dispersion trends of bacterial and algal communities across different stages of the two typhoons. **f** Comparative response trajectories of algae (top) and bacteria (bottom) across different typhoon events. Blue solid circles represent samples from the Prapiroon period, while red triangles represent samples from the Wipha period

During the Prapiroon event, the bacterial community was dominated by Pseudomonadota (mean relative abundance: 41.6%), followed by Actinomycetota (18.3%), Bacteroidota (17.9%), and Cyanobacteriota (16.3%) (Fig. S2a and Table S2). The phytoplankton community consisted primarily of Bacillariophyta (54.1%), Chlorophyta (24.7%), and Pyrrophyta (18.3%) (Fig. S2b and Table S3). During the Wipha event, the bacterial community exhibited a similar overall structure dominated by Pseudomonadota (52.9%), though the relative abundance of Bacteroidota was lower (10.2%) (Fig. 2a and Table S2). In contrast, the phytoplankton community displayed a distinct pattern, with Chlorophyta becoming dominant (56.9%), while the relative abundances of Bacillariophyta and Pyrrophyta declined to 32.3% and 8.8%, respectively (Fig. S2c and Table S3). Across the two typhoon events, the relative abundance of 15 bacterial phyla and 26 phytoplankton genera differed significantly. Relative abundances were higher during the Prapiroon event for 14 bacterial phyla and 14 phytoplankton genera, whereas the Wipha event showed higher relative abundances for only one bacterial phylum and 12 phytoplankton genera (Fig. 2b and Fig. S3).

Functional prediction via PICRUSt2 based on the KEGG database showed that both typhoon events shared similar predicted core functional profiles. At KEGG level 1, Metabolism was the dominant functional category (Prapiroon: 71.0%; Wipha: 70.93%), while at level 2, predicted functions were primarily represented by Carbohydrate metabolism (Prapiroon: 15.47%; Wipha: 15.34%) and Amino acid metabolism (Prapiroon: 15.05%; Wipha: 15.23%) (Fig. S4a–b). These results suggest that although typhoon events caused significant shifts in bacterial community composition and diversity, the predicted core functional profiles in Zhanjiang Bay remained relatively stable under disturbance.

Community diversity fluctuated across the sampling stages (Fig. 2c). Bacterial Shannon diversity followed a “decline-then-rebound”, whereas phytoplankton diversity displayed the opposite trend; both reached median extrema during stage 1 (Prapiroon: bacterial minimum = 1.44, phytoplankton maximum = 2.51; Wipha: bacterial minimum = 1.05, phytoplankton maximum = 2.25). Community composition differed significantly across stages for both typhoons (Fig. 2d and Fig. S5a–c). Notably, the within-group dispersion of bacteria and phytoplankton exhibited inverse trends (Fig. 2e), where convergence of one community coincided with divergence of the other. Overall, the two kingdoms displayed a synchronized yet inversely coupled successional pattern under disturbance.

Microbial community trajectories exhibited consistent directional patterns across both typhoon events. Bacterial and phytoplankton communities followed similar trajectories in ordination space, as indicated by high cosine similarity (bacteria: cos θ = 0.824, θ = 34.5°; phytoplankton: cos θ = 0.94, θ = 19.8°) (Fig. 2f). However, Prapiroon induced greater community displacement in ordination space (Table S4), with 2.38-fold (bacteria) and 2.53-fold (phytoplankton) higher cumulative trajectory lengths compared to Wipha, indicating a stronger spatial spread of community states across sampling stages.

### Performance evaluation of machine learning models

Α-diversity and β-dispersion describe complementary dimensions of community structure by reflecting within-community complexity and between-community divergence, respectively. Given the similar successional trajectories observed across both typhoons, datasets were integrated to quantify the negative correlation between bacterial and phytoplankton communities and to decipher the drivers of their coupled dynamics via multiple machine learning models. In the modelling framework, the explanatory matrix consisted of bacterial community composition and environmental variables, while the response matrix was defined as phytoplankton diversity metrics, with each observation corresponding to a sampling unit.

**Fig. 3:**
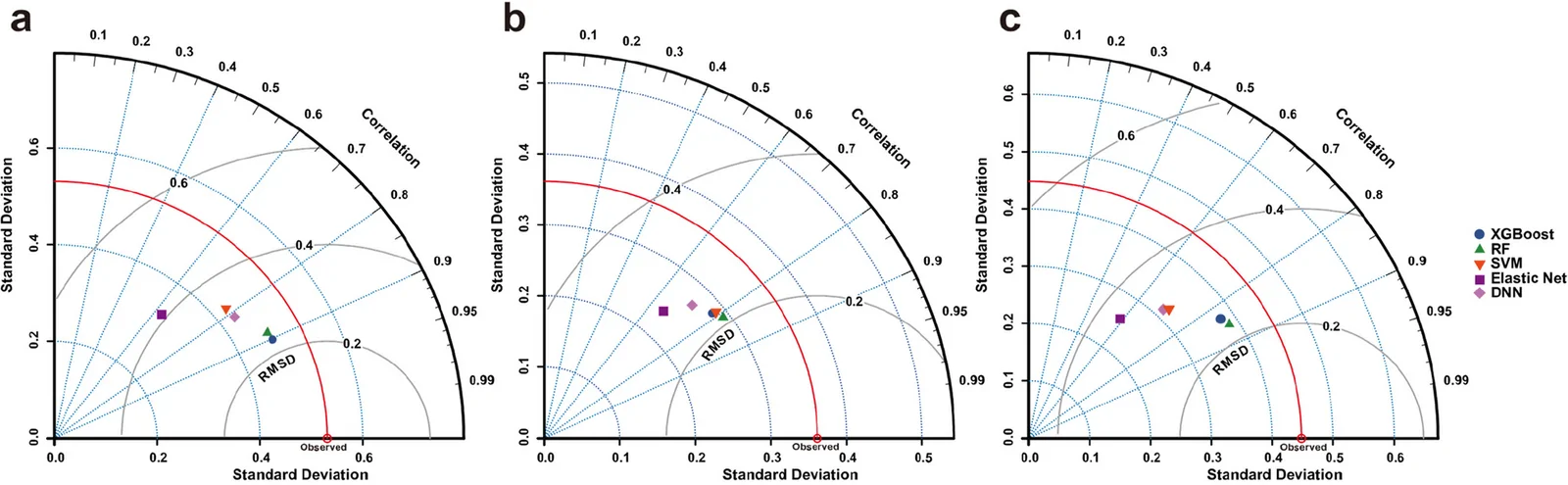
Evaluation of model generalization and predictive performance across different datasets. **a** Performance of different models on the Prapiroon test set. **b** Generalization of models trained on the Prapiroon training set when applied to the Wipha dataset. **c** Predictive performance on the test set after integrating all samples. Symbol positions indicate the standard deviation of model outputs and their correlation with observations. Red circles represent the standard deviation of observed values (Observed); the angular position of points relative to the circle corresponds to the Pearson correlation coefficient, and the radial distance from the circle indicates the root mean square deviation (RMSD)

Model fitting and predictive performance were first evaluated on the Prapiroon dataset. On the training set, XGBoost exhibited the best fit (R^2^ = 0.97, RMSE = 0.05), with the DNN also achieving high performance (R^2^ = 0.99, RMSE = 0.05), followed by RF, SVM, and ElasticNet (Fig. S6a). On the test set, XGBoost maintained the highest predictive accuracy (R^2^ = 0.81, RMSE = 0.23), followed by RF and SVM. In contrast, the DNN showed slightly reduced generalization, and ElasticNet performed poorly (R^2^ = 0.40, RMSE = 0.41) (Fig. 3a).

To evaluate the cross-event generalization, models were trained and tested on the Wipha dataset. On the training set, XGBoost (R^2^ = 0.98, RMSE = 0.05) and RF (R^2^ = 0.97, RMSE = 0.06) exhibited the best performance, followed by SVM, DNN, and Elastic Net (Fig. S6b). On the test set, RF slightly outperformed XGBoost (R^2^ = 0.66 vs. 0.63), while SVM and DNN showed intermediate performance, and Elastic Net remained the weakest (R^2^ = 0.43, RMSE = 0.27) (Fig. 3b).

After training on the combined dataset, XGBoost, RF, and DNN all yielded strong fits on the training set (R^2^ ≥ 0.95, RMSE ≤ 0.10), while SVM showed slightly lower performance (R^2^ = 0.95, RMSE = 0.10), and Elastic Net performed the worst (R^2^ = 0.56, RMSE = 0.30) (Fig. S6c). On the combined test set, RF achieved the best performance (R^2^ = 0.74, RMSE = 0.23), followed by XGBoost (R^2^ = 0.71, RMSE = 0.24), with SVM and DNN showing intermediate performance, and Elastic Net performing the weakest (R^2^ = 0.35, RMSE = 0.36) (Fig. 3c).

Overall, all machine learning models demonstrated robust capabilities in both fitting and prediction. Specifically, XGBoost provided the best performance for the Prapiroon dataset, while RF performed best for the Wipha and combined datasets. Compared with linear models (ElasticNet), non-linear approaches—particularly RF and XGBoost—consistently achieved superior performance across all datasets, whereas the deep learning model (DNN) exhibited signs of mild overfitting.

### Identification of key drivers and their stage-specific effects

**Fig. 4:**
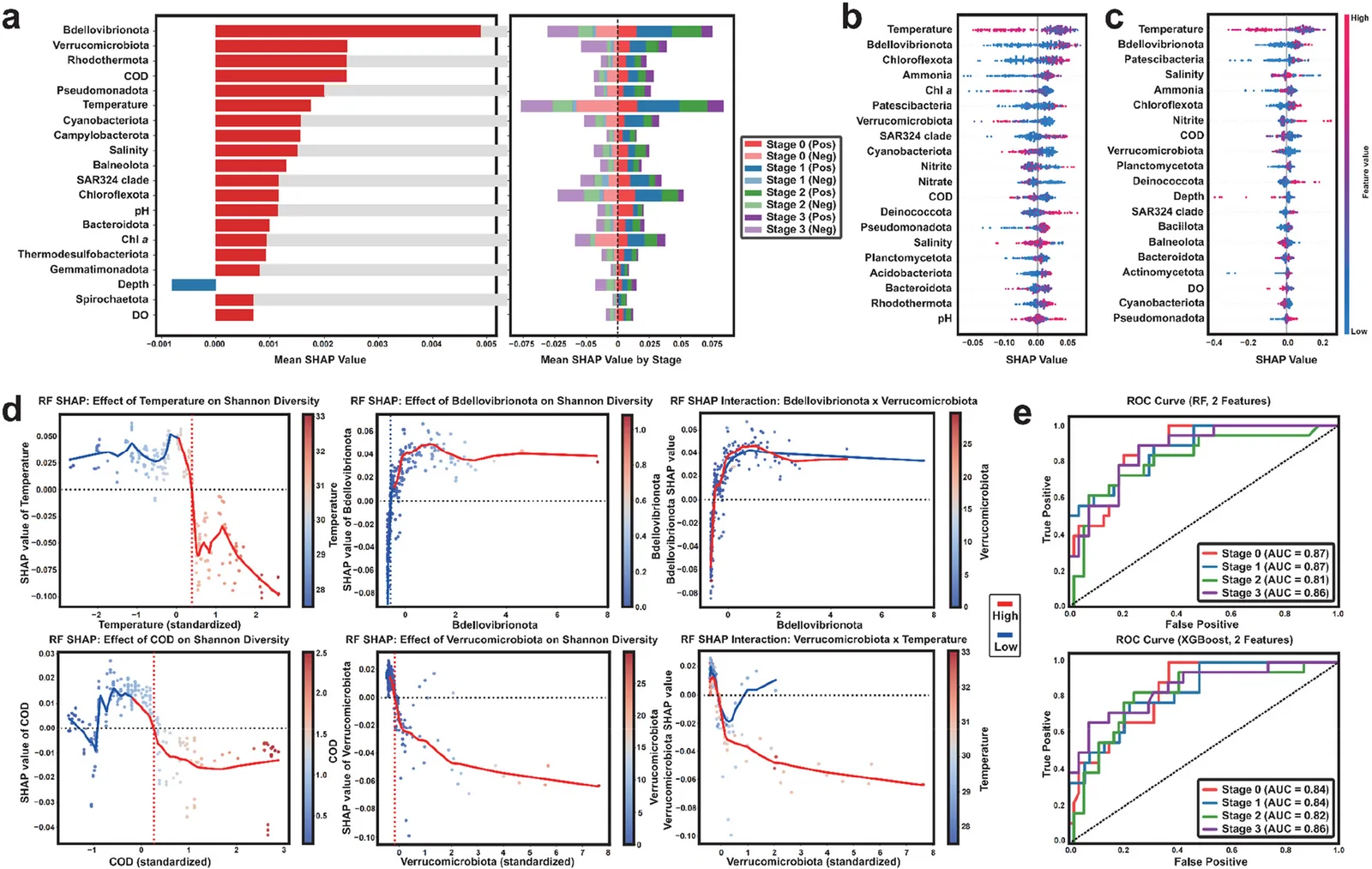
Revealing model-identified key features and temporal classification performance. **a** Stage-specific contribution trends of the top 20 key predictive features identified by RF. Each bar represents the mean SHAP value of a feature. Solid red bars indicate positive contributions, and semi-transparent blue bars indicate negative contributions, sorted by absolute contribution. **b–c** Top 20 key predictive features and their contribution directions identified by RF (left) and XGBoost (right). Each point represents a feature’s SHAP value, and color denotes the standardized feature value. Points are ordered by SHAP value ranges. Positive SHAP values indicate a positive contribution to phytoplankton diversity (increasing predicted values), while negative values indicate the opposite. **d** Single-feature and interaction effects identified by RF. LOWESS low-pass filtered curves for samples with feature2 above the median (red) and below the median (blue). **e** Multi-stage classification performance of RF and XGBoost classifiers using Temperature and Bdellovibrionota as features. Black dashed lines indicate the random classification baseline (AUC = 0.5)

After validating model performance, SHAP analysis was performed on the combined dataset using the top-performing architectures to quantify feature contributions and identify key drivers. Among the top 20 predictors identified by both RF and XGBoost, Bdellovibrionota (ranked 1 st by RF and 2nd by XGBoost) emerged as the most influential predictor, followed by Verrucomicrobiota (Fig. 4a and Fig. S7). Among abiotic variables, Temperature (ranked 6th by RF and 1 st by XGBoost) and COD (ranked 3rd by RF and 4th by XGBoost) were also identified as important drivers. Notably, the stage-specific shifts in temperature’s contributions closely mirrored the ecological effects of Bdellovibrionota, exhibiting a highly synchronized pattern (Fig. 4a and Fig. S7). Before the typhoons (Stage 0), both factors exhibited relatively strong negative effects on phytoplankton diversity (Temperature: positive vs. negative effects = 9.5% vs. 20.39%; Bdellovibrionota: 11.5% vs. 13.25%). In the immediate aftermath (Stage 1), their contributions reversed markedly, with positive effects increasing sharply (Temperature: 21.07% vs. 2.01%; Bdellovibrionota: 21.4% vs. 1.8%). During the recovery phase (Stage 3), this promoting effect gradually diminished, eventually reverting to inhibitory patterns similar to those observed in Stage 0 (Temperature: 7.83% vs. 15.77%; Bdellovibrionota: 6.55% vs. 18.77%).

Stage-wise aggregation of the top 20 features confirmed that overall contribution patterns aligned with these trends. At Stage 0, positive and negative contributions were relatively balanced (15.51% vs. 12.46%), indicating a pre-disturbance equilibrium. However, this balance was disrupted at Stage 1, when positive contributions increased (18.24%) and negative contributions sharply decreased (3.85%), suggesting a strong shift in phytoplankton regulatory drivers under typhoon disturbance. This gap narrowed during Stage 2 (15.50% vs. 10.52%), as the system entered an adjustment phase. By Stage 3, negative contributions (16.46%) exceeded positive ones (7.47%), exerting a clear inhibitory effect on phytoplankton diversity during the later recovery phase. The mechanistic details of the most influential features are further elucidated in Fig. 4b–c. For temperature and COD, the positive SHAP contribution regions (right of the zero axis) were predominantly occupied by low-value samples (blue points), indicating that lower levels of these abiotic factors favored higher phytoplankton diversity post-disturbance. Conversely, high-abundance samples of specific bacterial taxa, such as Bdellovibrionota, were more frequently clustered in the positive contribution regions. This suggests that the proliferation of these bacterial groups played a pivotal role in supporting phytoplankton diversity during the successional process.

Further SHAP dependence analysis (Fig. 4d) delineated the specific relationships between key predictors and phytoplankton diversity. Both temperature and COD exhibited clear threshold-based inhibitory effects: phytoplankton diversity declined when temperature exceeded 30.76 °C, or when COD levels surpassed 1.16 mg/L. Among biotic factors, Bdellovibrionota exerted a positive effect once its relative abundance reached a threshold of 0.02%, whereas Verrucomicrobiota transitioned to an inhibitory role at abundances exceeding 1.02%. Additionally, no significant interaction effects were detected between Bdellovibrionota and other key variables (Verrucomicrobiota, temperature, or COD), suggesting its ecological impact was primarily driven by main effects (Fig. S8). In contrast, interactions between Verrucomicrobiota and environmental gradients (temperature and COD) became evident only after crossing the 1.02% abundance threshold. This indicates that the direction and magnitude of Verrucomicrobiota’s influence are condition-dependent and vary along environmental gradients, representing a typical "threshold-mediated" response. Overall, the stage-specific mean variations in key drivers (e.g., decreased Temperature and increased relative abundance of Bdellovibrionota during Stages 1–2) were consistent with the directional changes in phytoplankton Shannon diversity across stages. (Fig. S9 and Table S5). Consistent patterns were also confirmed through XGBoost feature effect analysis (Fig. S10).

Given the representative stage-specific contributions of temperature and Bdellovibrionota, we constructed a RF classifier based exclusively on these two variables to distinguish the four typhoon stages. ROC curve analysis demonstrated high discriminative power, with Area Under the Curve (AUC) values of 0.87 for both Stage 0 and Stage 1, 0.86 for Stage 3, and 0.81 for Stage 2 (Fig. 4e). The confusion matrix further indicated that misclassifications occurred primarily between adjacent stages (Stage 0 vs. 1, and Stage 2 vs. 3; Table S6). In terms of classification metrics, precision and recall ranged from 0.5 to 0.72, with F1 scores (0.56–0.61) reflecting a balanced performance across stages (Table S7). Similar trends were observed for XGBoost, although its overall discriminative ability was slightly lower than that of RF (Fig. 4e; Tables S8–S9). Collectively, the ROC curves indicate that both RF and XGBoost could effectively capture the discriminative signals of temperature and Bdellovibrionota abundance across stages, suggesting that RF can reliably distinguish early stages of the typhoon (Stage 0–1) from the later recovery stage (Stage 3), whereas its performance in identifying samples during the typhoon (Stage 2) is relatively weaker. Importantly, even using only these two key features, the models were able to capture the stage-specific dynamics of phytoplankton communities under typhoon disturbance.

### Collapse and coupled recovery of Bacteria-Phytoplankton networks under typhoon disturbance

**Fig. 5:**
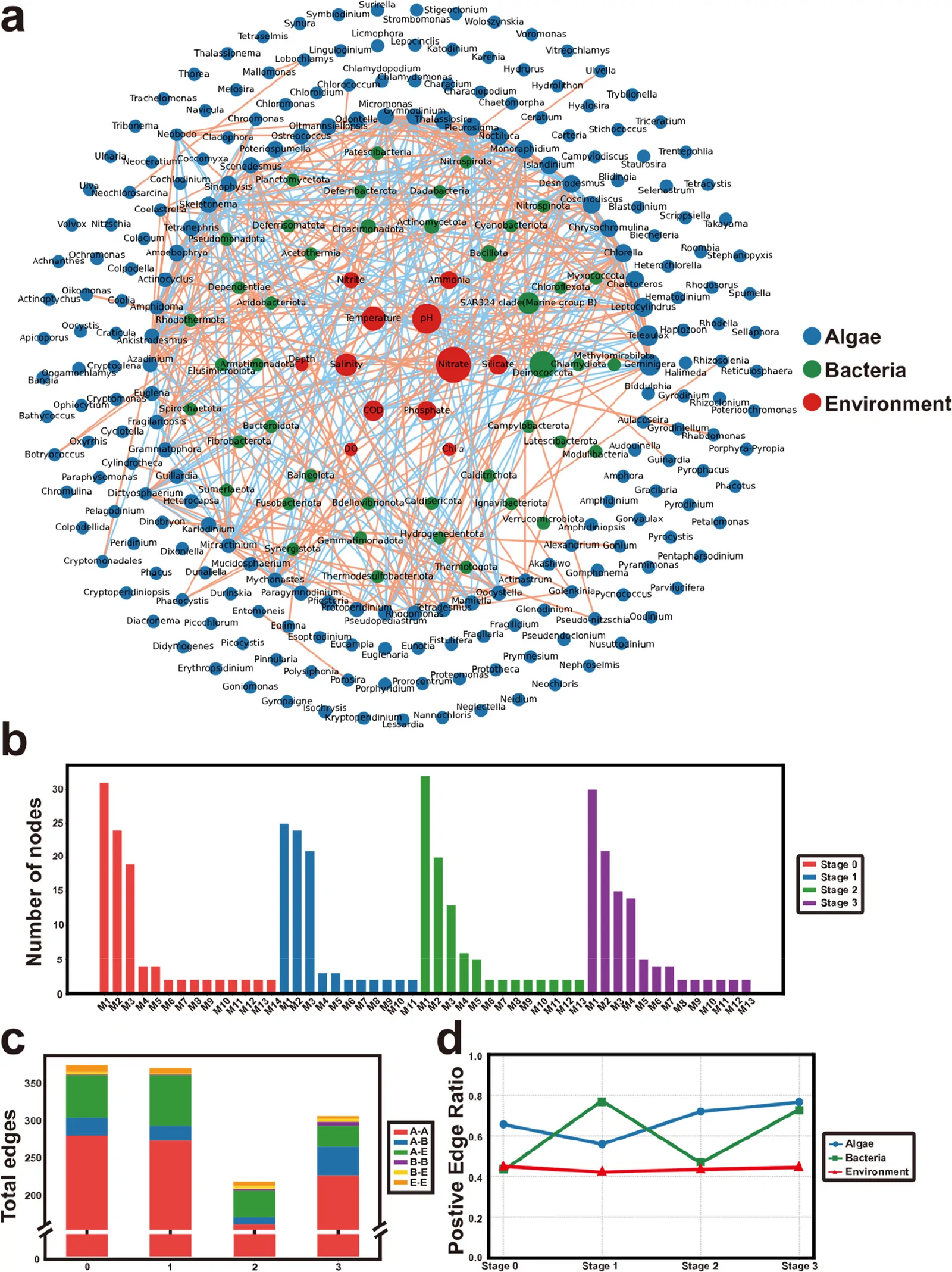
**a** Overview of the bacteria-phytoplankton association network during Stage 1. Positive associations (orange) indicate taxa co-occurrence and potential synergistic relationships, whereas negative associations (blue) suggest antagonistic relationships. **b** Network structures show clear dynamic changes across stages. Using Louvain community detection, networks were partitioned into modules, each representing a cluster of more tightly associated features. Colors are used to distinguish different stages. **c** Proportion of weighted edges for different interaction types. Colors for different interaction types are shown in the legend. **d** Temporal trends in the proportion of positive correlation edges among the three feature types across different stages, with colors and symbols indicating different feature types

To resolve the association structure of the microbial community, bacteria-phytoplankton ecological association networks were constructed for the four successional stages (Fig. 5a and Fig. S11). The results showed that Stage 0 possessed the most robust structure, comprising 14 modules, with large core modules (Module 1: 31; Module 2: 24; Module 3: 19) forming a tightly connected and stable interaction pattern (Fig. 5b). In Stage 1, the network noticeably contracted to only 11 modules. The number of nodes and the proportion of positive interactions reached their minimum levels (Nodes: 88; Positive edge ratio: 0.56), while the average path length shortened to 2.85. These shifts indicated network disintegration under intense disturbance, characterized by loosened local connectivity and an increase in negative associations (Table S10). Stage 2 featured both large and numerous medium-to-small modules (Module 1: 32). During this phase, the number of weighted edges (215) and the clustering coefficient (0.396) dropped to their lowest values, while the network radius peaked (4.67). These topological changes suggest that interactions were concentrated on a few key nodes while local structures remained highly fragmented, representing a transient and unstable phase of network reorganization. By Stage 3, the dominant modules expanded again (Module 1: 30; Module 2: 21; Module 3: 15). The concurrent increase in node count, positive edges, and weighted edges indicates that the network had regained complexity and was approaching a new steady state.

The fluctuations of interaction edges further reflected the differential responses of various taxa (Fig. 5c; Table S11). Within-type interactions showed that algal-algal (A-A) interactions exhibited the largest absolute fluctuations (SD: 73.5) while remaining relatively stable (CV: 24.3%). In contrast, bacteria-bacteria (B-B) interactions exhibited the highest relative variability (CV: 50.3%) and increased sharply from Stage 2 to Stage 3 (15 to 48), suggesting that intra-group bacterial interactions were the most sensitive to typhoon disturbance.

Among cross-type interactions, algal–bacteria (A-B) interactions exhibited the largest relative fluctuations (SD: 16.14; CV: 55.7%). A-B interactions declined to their lowest level at Stage 2 and then showed a pronounced rebound at Stage 3 (from 11 to 50), representing the strongest recovery among all interaction types. Moreover, although the positive edge ratio of environmental factors exhibited minimal fluctuations (0.42–0.45), its temporal trend mirrored that of phytoplankton entirely (Fig. 5d), highlighting the tight coupling between algal community structure and environmental changes.

Overall, the typhoon events drove the bacteria–phytoplankton network through a dynamic succession characterized by a "steady state–collapse–reorganization–re-stabilization" trajectory. While bacterial communities were highly sensitive to the disturbance, they achieved reconstruction through coupled synergistic interactions with the phytoplankton, eventually steering the system toward a new equilibrium.

## Discussion

### Unified biological response patterns to heterogeneous disturbances

Our results indicate that although Typhoons Prapiroon and Wipha differed substantially in stochastic physical attributes such as track and energy intensity, the microbial community responses they induced exhibited highly consistent coupling dynamics. Prapiroon did not directly pass over Zhanjiang Bay and had relatively lower wind intensity; however, its passage over Hainan Island, characterized by a limited land area, likely reduced energy dissipation, allowing the typhoon to retain considerable strength upon reaching the study area. In contrast, Wipha exhibited stronger wind intensity at landfall and a trajectory closer to Zhanjiang Bay, but experienced greater energy dissipation along its longer overland path, resulting in a weaker overall impact on the bay. These contrasting pathways led to distinct hydrodynamic responses: when a typhoon passes to the left of Zhanjiang Bay, the Coriolis effect induces a clockwise circulation that drives offshore seawater into upstream estuarine regions, suppressing riverine export and promoting the formation of frontal structures, thereby enhancing the retention of nutrients and plankton within the bay [40, 41]. In contrast, when the track lies to the right of the bay (as in Wipha), the hydrodynamic response follows an opposite pattern. This asymmetry likely explains the stronger vertical mixing and sediment resuspension induced by Prapiroon, which contributed to more pronounced community succession and a higher relative abundance of rare bacterial phyla such as Bdellovibrionota and Verrucomicrobiota.

However, despite differences in the intensity of physical disturbance and hydrodynamic responses among typhoon events, the overall consistency in microbial community responses suggests that typhoon disturbances may trigger a predictable trajectory of community reassembly shaped jointly by environmental filtering and biotic interactions, rather than simple stochastic fluctuations. This feature closely aligns with the phenomenon of “directional recovery” commonly observed in aquatic ecosystems following strong disturbances [10, 42]. Accordingly, this potential successional pathway reflects the dominant role of environmental disturbance in governing microbial community dynamics, whereas differences among typhoon events are primarily expressed in the magnitude of displacement along the reassembly trajectory rather than in its direction. Such a positive relationship between “disturbance dose” and the magnitude of ecological response is also theoretically consistent with patterns observed under other extreme events, including droughts and heatwaves [43, 44].

Notably, the predicted core functions of the bacterial community remained relatively stable under both typhoon disturbances. This decoupling between compositional changes and functional stability suggests a potential degree of functional redundancy in the bacterial community of Zhanjiang Bay, which may contribute to buffering ecosystem responses to environmental disturbances and maintaining the continuity of key biogeochemical processes [45, 46].

### Multilevel integrated regulation of phytoplankton communities driven by typhoon disturbances

Our results indicate that the stage-specific shifts in phytoplankton diversity under typhoon disturbance arise from the dynamic coupling between abiotic stressors—such as temperature and chemical oxygen demand (COD)—that impose direct physiological stress on algal cells, and ecological regulation mediated by key functional bacterial groups, particularly Bdellovibrionota. As the most influential abiotic driver identified by the model, temperature may primarily exert its effects through cellular physiological pathways. Elevated temperature stress can activate calcium ion signaling pathways in algal cells, disrupt photosynthetic processes, and potentially induce programmed cell death, thereby directly filtering community composition [47]. Meanwhile, high COD, indicative of elevated organic loading, enhances microbial respiration. The CO₂ released during organic matter degradation alters the carbonate system and leads to localized water acidification, forcing phytoplankton to expend additional energy to maintain intracellular pH homeostasis and thereby indirectly suppressing overall community diversity [48, 49].

In contrast, biotic regulation of phytoplankton diversity is more prominently manifested through top-down effects mediated by food-web structure. Although the mean relative abundance of Bdellovibrionota remained below 0.1%, members of this group have been reported as obligately predatory Gram-negative bacteria capable of attaching to, invading, and lysing heterotrophic bacteria that may compete with phytoplankton for nutrients. Through these previously described ecological traits, Bdellovibrionota may indirectly influence nutrient availability and microbial interactions within the system. Meanwhile, bacterial predation has been suggested to contribute to the release and recycling of dissolved nutrients, potentially shaping the local microenvironment and indirectly affecting phytoplankton-associated ecological conditions [50]. During Stage 1, the pronounced increase in the positive contribution of Bdellovibrionota suggests that physical disturbance may activate its ecological function. By rapidly “pruning” the bacterial community, Bdellovibrionota creates a transient window characterized by low competitive pressure and high nutrient availability, thereby facilitating the recovery of phytoplankton—particularly r-strategist species—and ultimately driving the rapid increase in phytoplankton diversity.

In summary, our study supports a stage-specific, multi-level regulatory pattern. Typhoons initially reset the environment—such as enhancing vertical mixing, resuspending Bdellovibrionota, and inducing surface cooling—thereby triggering switches in phytoplankton community composition. As the disturbance dissipates, sea-surface temperatures rebound and environmental quiescence strengthens selective pressures, ultimately suppressing diversity in the later stages and driving the system toward a new stable state [51, 52]. By integrating our quantitative findings with known cellular mechanisms and ecological processes, this work paves the way toward a better understanding of ecosystem responses to acute climatic disturbances.

### Asynchronous responses of bacteria and phytoplankton in cross-kingdom homeostatic regulation

Our study further supports that statistical associations between bacteria and phytoplankton shift from a competitive equilibrium to a disturbance-triggered pattern of negative ecological coupling. Under stable environmental conditions, phytoplankton and bacteria exhibit a continuum of “mutualistic-competitive” relationships. Phytoplankton, as primary producers, and bacteria, primarily decomposers, share the same nutrient pool, competing for dissolved inorganic nutrients such as nitrogen and phosphorus [53, 54]. Meanwhile, they form a complex interaction network through processes such as the release of dissolved organic matter by phytoplankton and the remineralization of nutrients by bacteria [55]. In the calm pre-typhoon period, these nutrient- and chemically-mediated interactions may reach a dynamic equilibrium.

Typhoons, as high-intensity physical disturbances, can instantaneously disrupt this homeostasis. Notably, the resource pulses triggered by such disturbances do not constitute a “fair dividend” for bacteria: the large influx of inorganic nutrients released by a typhoon is typically first assimilated by primary producers such as phytoplankton and rapidly converted into biomass, whereas bacterial growth largely depends on the lagged secondary resource supply, including dissolved organic carbon (DOC) and particulate organic matter (POM) released by phytoplankton [56]. At the same time, the intense disturbance amplifies environmental filtering and community homogenization processes, imposing stronger selective pressure on sensitive bacterial taxa and leading to a sharp decline in bacterial diversity [57]. In contrast, phytoplankton, as opportunistic taxa highly sensitive to nutrient pulses, can respond rapidly and occupy vacant ecological niches, exhibiting an increase in diversity over short time scales. During the post-typhoon recovery phase, bacterial and algal communities exhibit asynchronous successional trajectories, which may reflect a potential self-regulatory pattern. While both recover concurrently, they appear to adopt distinct functional strategies, potentially reducing direct competition for limited resources. This allows the system to sequentially rebuild primary production dominated by phytoplankton and decomposition and remineralization functions dominated by bacteria, promoting the gradual restoration of biogeochemical cycling processes [58].

In addition, rare taxa within bacterial communities and the associated diversity reservoir may act as an “ecological insurance,” not only enhancing the stability of bacterial communities under environmental fluctuations but also radiating this stability to other functional groups, such as phytoplankton, via cross-taxon interactions and biogeochemical cycling. In this process, the rare biosphere often plays a critical yet overlooked regulatory role, providing an important safeguard for ecosystem resilience and maintaining community productivity and key ecological functions under extreme disturbances [59–61].

### Application value of artificial intelligence in ecological management

This study not only supports bacteria–phytoplankton associations but also explores the potential of integrating these insights into ecosystem prediction. Previous research has shown that typhoon disturbances often trigger harmful algal blooms (HABs), which essentially represent a process of community reassembly, with community diversity frequently changing during or around HAB events [62]. During bloom development, dominant phytoplankton or algal species can rapidly proliferate, driving community structure toward simplification, while diversity and bloom occurrence exhibit complex high-dimensional relationships—patterns that our study similarly captures. Our results indicate that algal diversity follows an initial increase followed by a decline after typhoon events, with diversity suppression during the later stages being more pronounced in the Prapiroon event, suggesting a higher likelihood of HAB occurrence following Prapiroon. Moreover, the Wipha event serves as an external validation, demonstrating the generalization capability of machine learning models in recognizing high-dimensional features. The identification of ecological fingerprints through threshold-based detection and stage classification further provides a basis for pinpointing critical intervention windows to prevent HAB formation. Taken together, these findings highlight the potential application of artificial intelligence in predicting algal diversity dynamics and HAB occurrences, as well as its promise for quantifying the stochasticity of typhoon-induced blooms.

We also acknowledge certain limitations. The features identified by machine learning models represent conditional importance relative to the predefined response variables; thus, they reflect correlations among effective drivers within specific ecological contexts rather than absolute causal hierarchies (including relationships inferred from ecological association networks). The underlying mechanisms driving ecosystem dynamics still require further validation through controlled experiments.

For future work, we propose several directions. First, sampling design should better account for tidal dynamics. Second, metabarcoding-based functional inference has inherent limitations, and integration with metagenomic approaches would improve the accuracy of ecosystem functional interpretation. Finally, incorporating a negative control dataset from the same period but without typhoon influence would further strengthen causal inference and enhance interpretability.

## Conclusion

By using two typhoon events with distinct physical disturbance characteristics as natural contrasts, this study provides insights into the predictability of microbial community responses under intense disturbance regimes in the Zhanjiang Bay system, while broader applicability to other ecosystems remains to be further tested. Our results suggest that typhoon disturbances not only induce stochastic fluctuations but may also be associated with a directional pathway of community reassembly jointly shaped by environmental filtering and biotic interactions. During the early phase of typhoon impact, bacterial communities appeared to experience stronger environmental filtering, whereas phytoplankton communities acted as opportunistic responders benefiting from typhoon-induced nutrient pulses. During the recovery phase, these two communities may have occupied functionally offset niches, collectively contributing to the buildup of ecological resilience. Building on this framework, we further identified the rare bacterial lineage Bdellovibrionota and Temperature as core cross-taxonomic regulatory axes, which also serve as ecological fingerprints characterizing disturbance time windows. These findings highlight the unique strengths of artificial intelligence in deciphering high-dimensional ecological processes and predicting disturbance responses, while revealing rare bacterial taxa as radiating “ecological fuses” that confer ecosystem insurance.

Overall, our study highlights the successional trajectories and complex patterns of microbial community dynamics under ecological disturbance, emphasizing the importance of these findings for biodiversity conservation.

## Supplementary Information

Supplementary Material 1.

## Data Availability

The raw sequencing data have been deposited in the NCBI Sequence Read Archive (SRA) under BioProject accession number PRJNA1455817.

## Abbreviations

Chl *a*: Chlorophyll *a*
COD: Chemical oxygen demand
BH: Benjamini-Hochberg method
RMSE: Root mean square error
RF: Random forest
XGBoost: EXtreme gradient boosting
SVM: Support vector machine
DNN: Deep neural network
SHAP: SHapley additive exPlanations
AUC: Area under the curve
