# Supplementary material for: From chaos to order: cross-kingdom coordination buffers microbial communities against typhoon impacts
Source: BMC Microbiol. 2026 Jul 11;26:836. doi: 10.1186/s12866-026-05387-9 (PMC13602602; doi:10.1186/s12866-026-05387-9)
Supplement: Supplementary file 1 [file 12866_2026_5387_MOESM1_ESM.pdf]

Supplementary Figures

Supplementary Figure S1

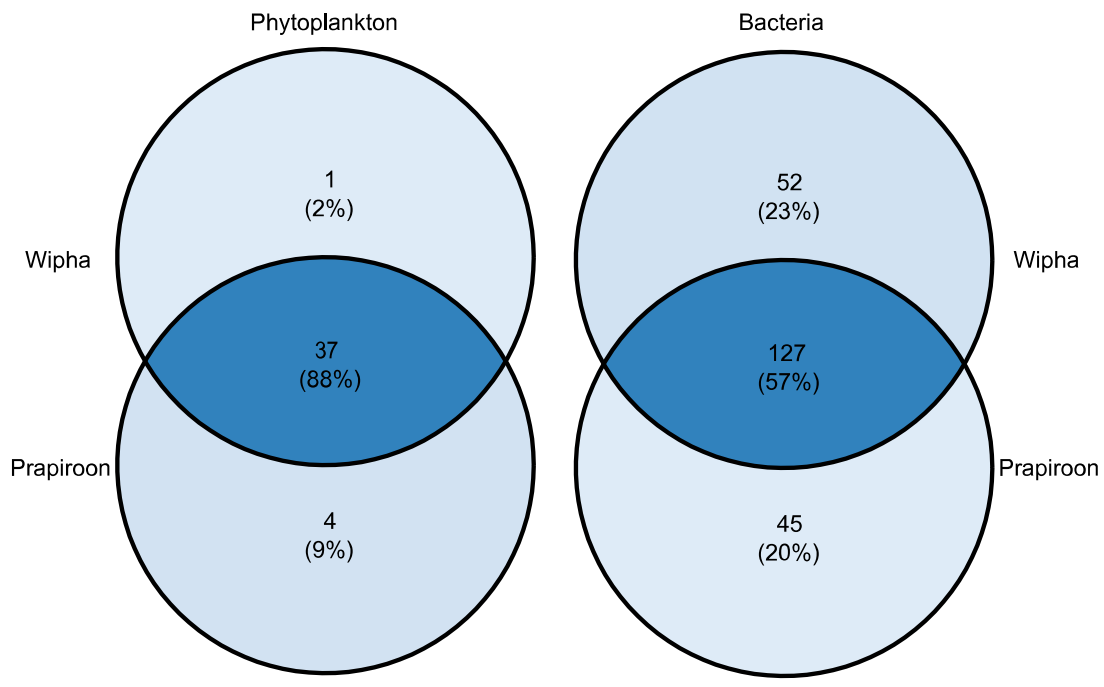

Fig. S1. Comparison of bacteria-specific phyla and phytoplankton-specific genera across different typhoon periods.

Supplementary Figure S2

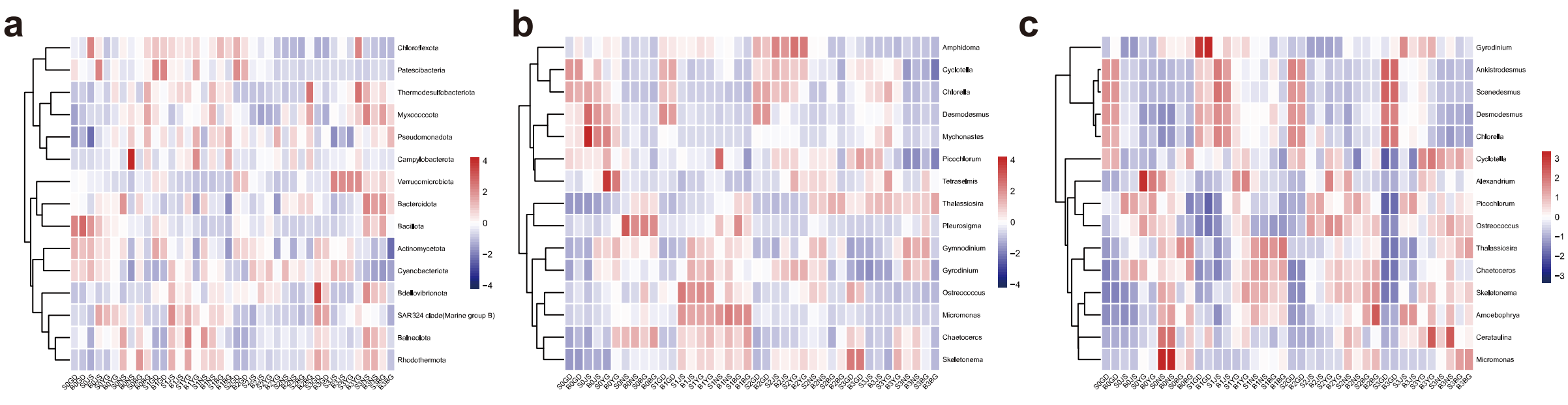

Fig. S2. **a** Mean relative abundance of the top 15 bacterial phyla during Typhoon Prapiroon (3 technical replicates). **b** Mean relative abundance of the top 15 algal genera during Typhoon Prapiroon (3 technical replicates). **c** Mean relative abundance of the top 15 algal genera during Typhoon Wipha (3 technical replicates). Values were Z-score standardized to highlight compositional differences across samples.

Supplementary Figure S3

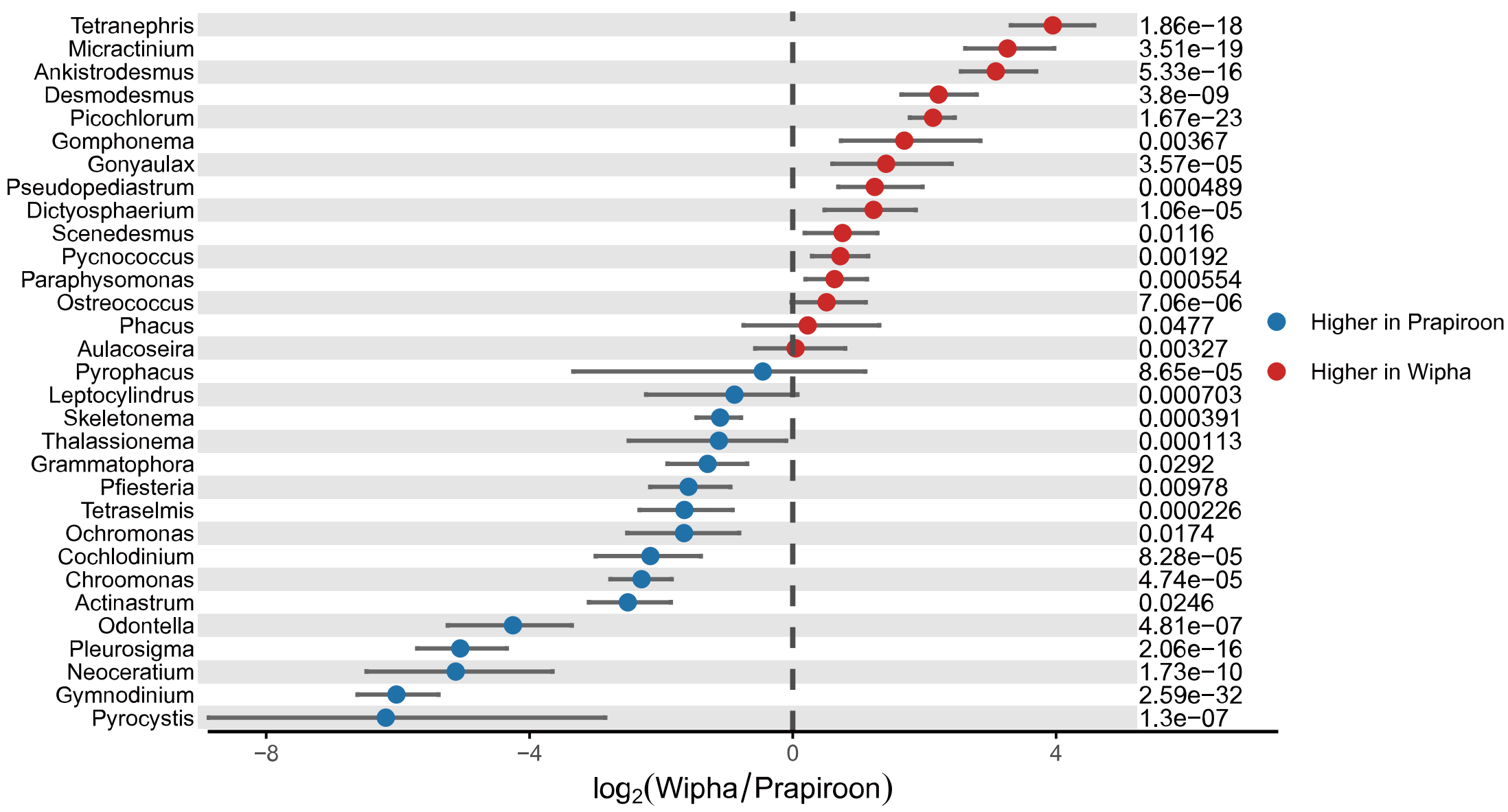

Fig. S3. Differential relative abundance of algal genera between the two typhoons. Values on the right represent adjusted p-values from Wilcoxon rank-sum tests.

Supplementary Figure S4

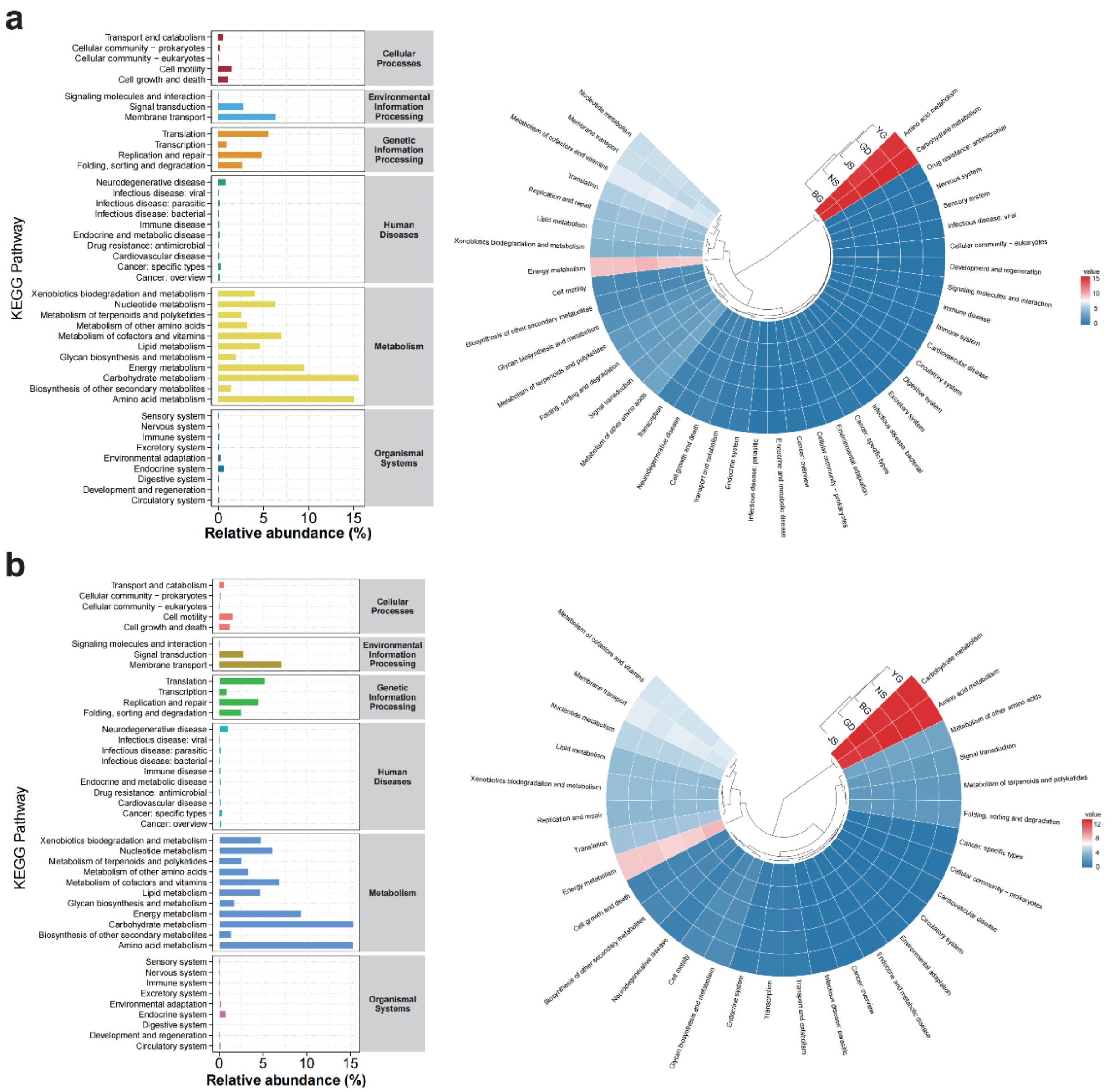

Fig. S4. **a** PICRUSt2-based functional predictions of bacterial communities during Typhoon Prapiroon using the KEGG database (mean NSTI = 0.158 ± 0.025 SD, n = 120). **b** PICRUSt2-based functional predictions of bacterial communities during Typhoon Wipha using the KEGG database (mean NSTI = 0.139 ± 0.029 SD, n = 120).

Supplementary Figure S5

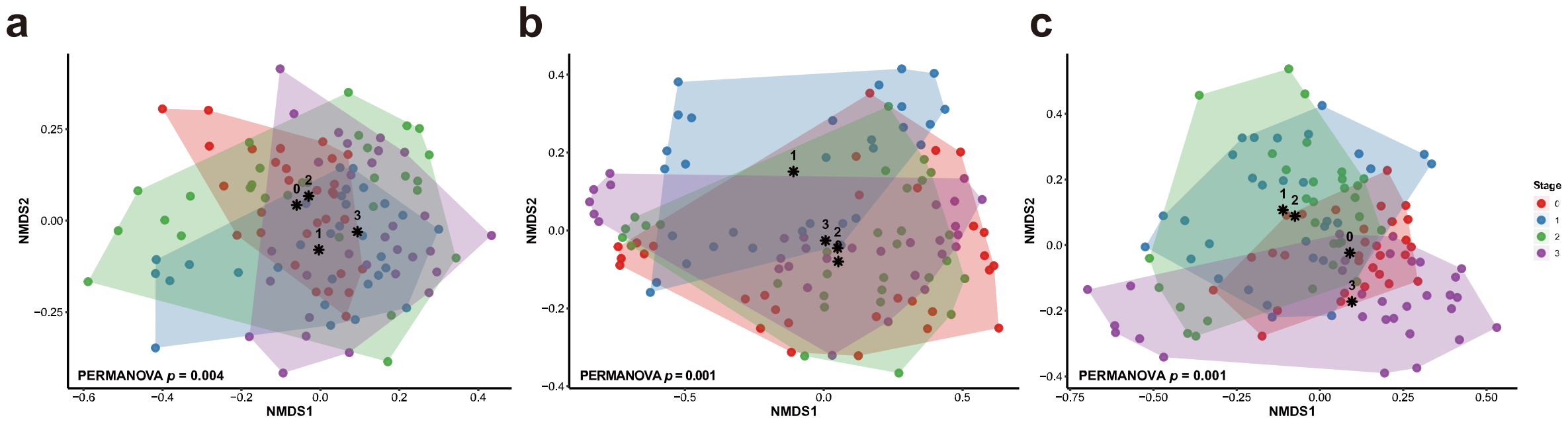

Fig. S5. **a** Stage-wise distribution differences of bacterial communities during Typhoon Prapiroon. **b** Stage-wise distribution differences of algal communities during Typhoon Wipha. **c** Stage-wise distribution differences of algal communities during Typhoon Prapiroon.

Supplementary Figure S6

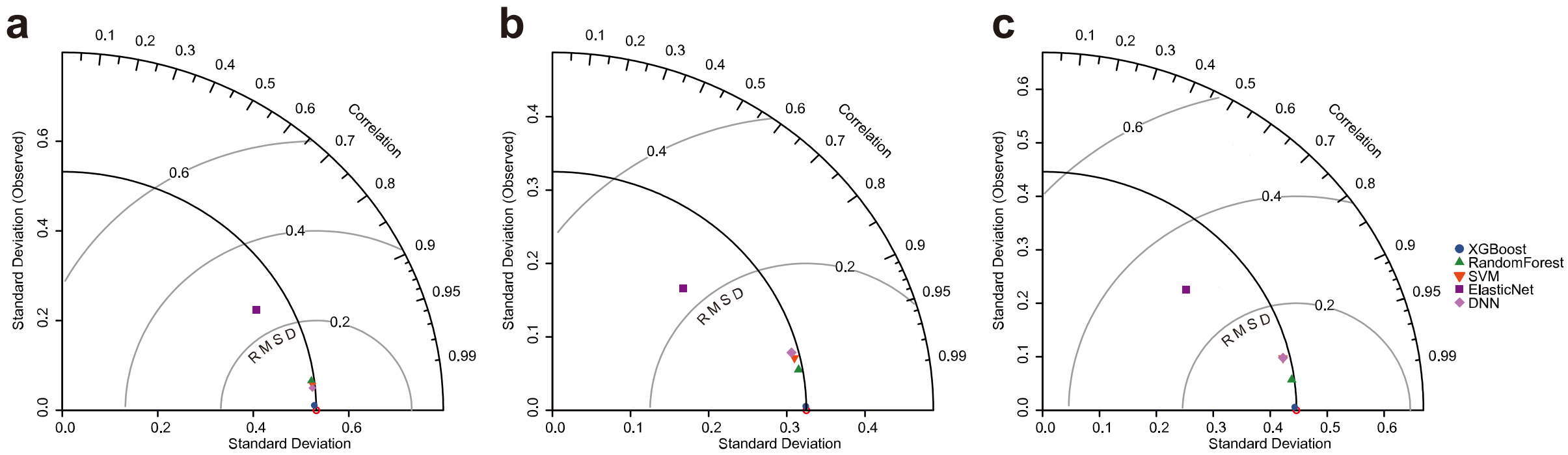

Fig. S6. **a** Performance of different models on the Prapiroon training set. **b** Performance of different models on the Wipha training set. **c** Training fit after integrating all samples.

Supplementary Figure S7

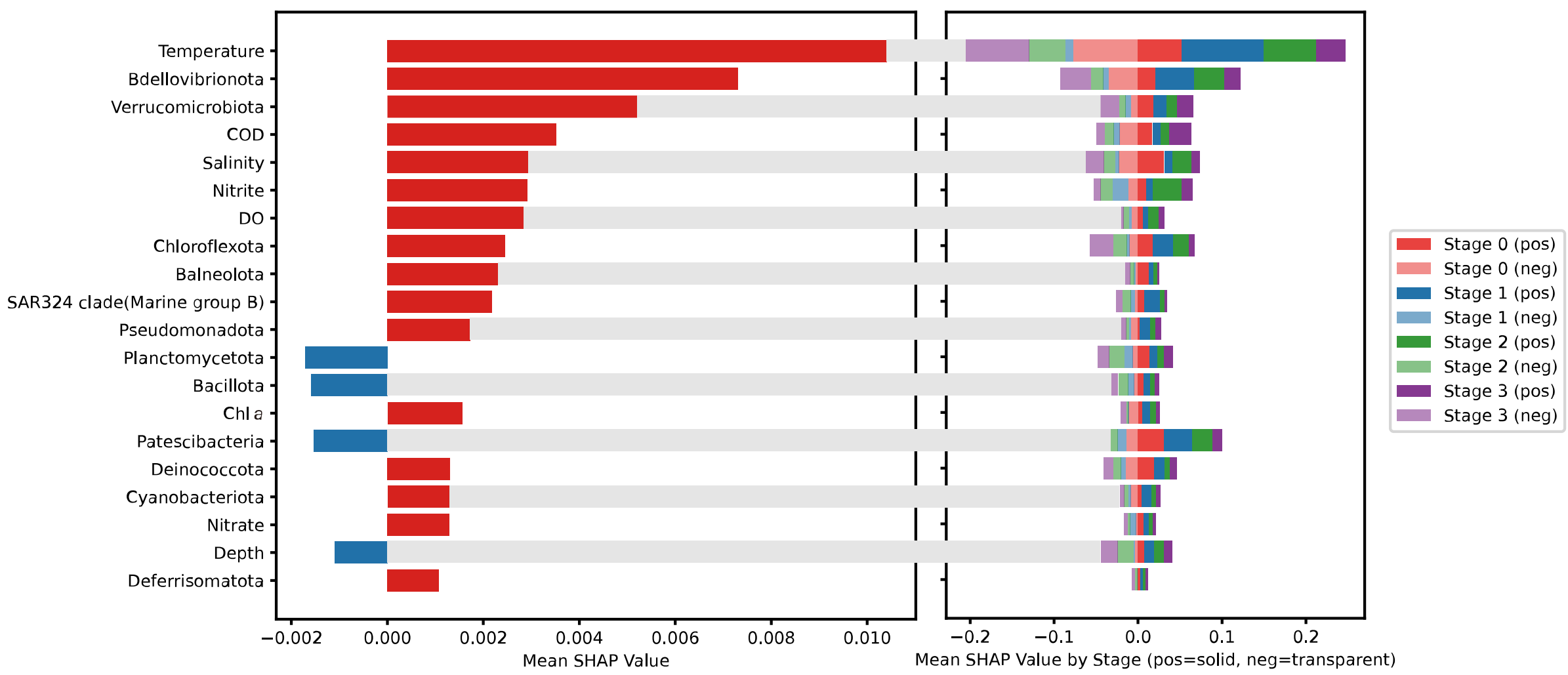

Fig. S7. Stage-wise contribution trends of the top 20 key predictive features identified by XGBoost.

Supplementary Figure S8

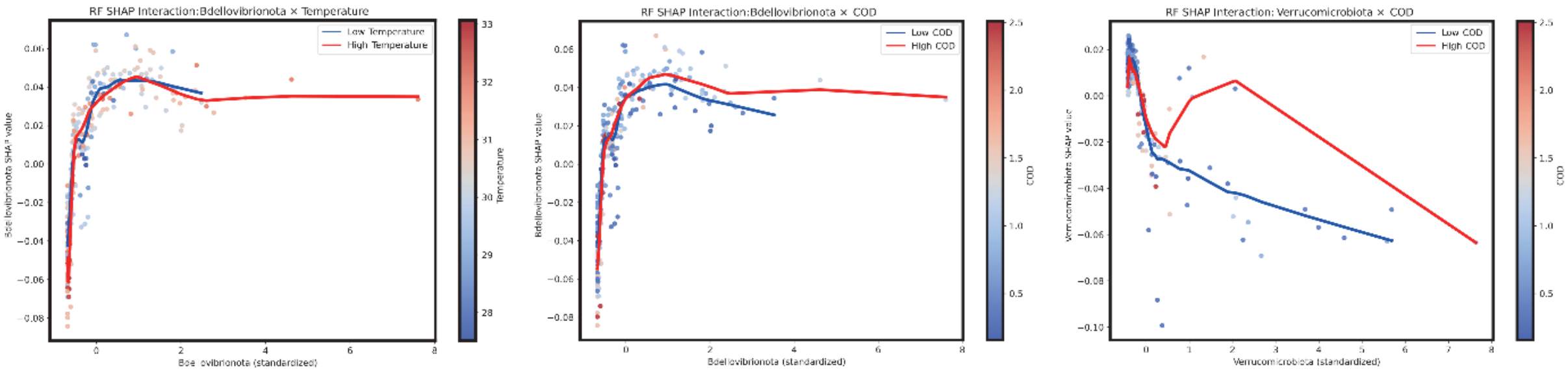

Fig. S8. RF-based identification of interactions between different factors.

Supplementary Figure S9

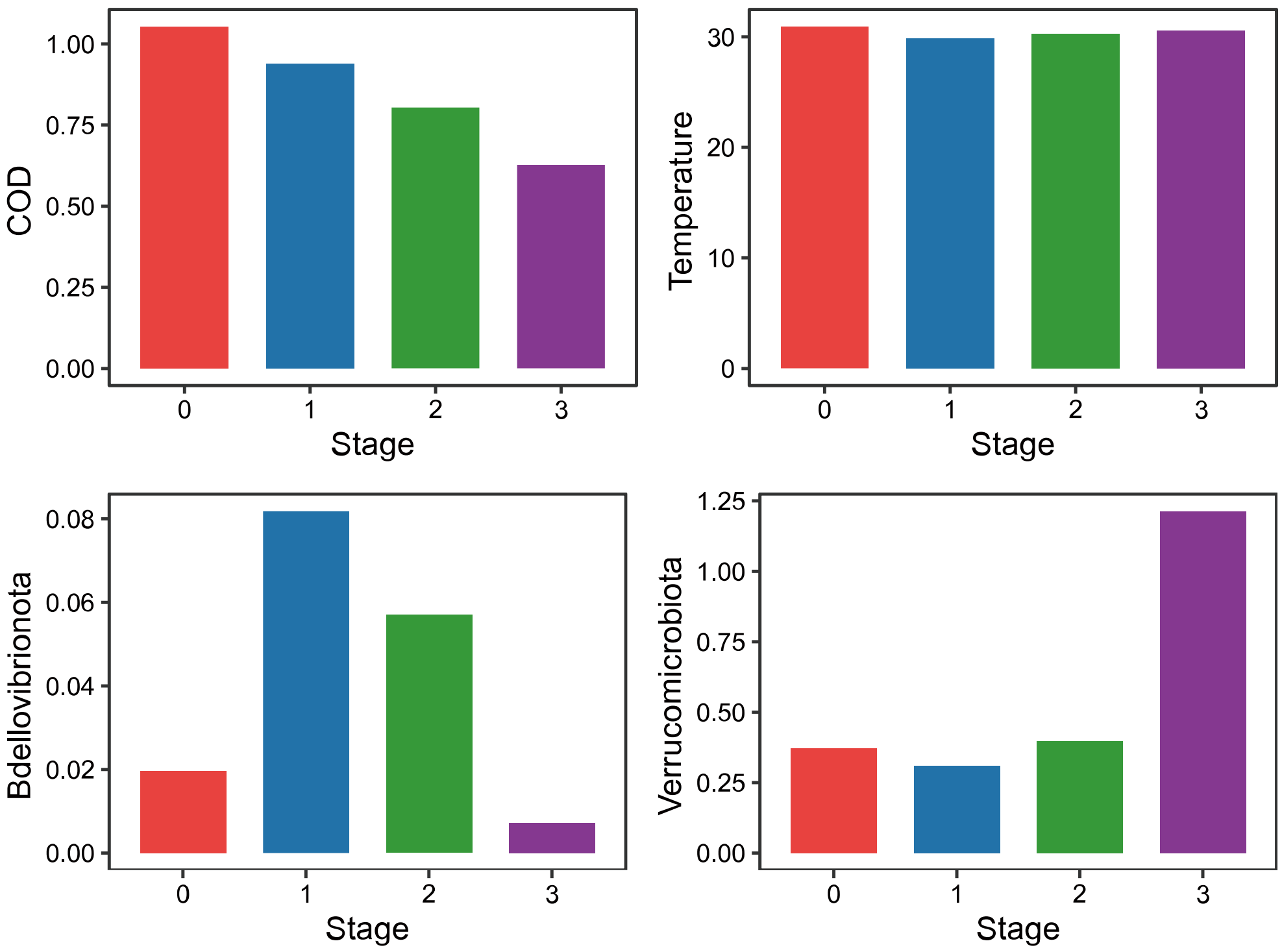

Fig. S9. Stage-wise mean changes in key driving features, calculated by pooling samples from both typhoon events.

Supplementary Figure S10

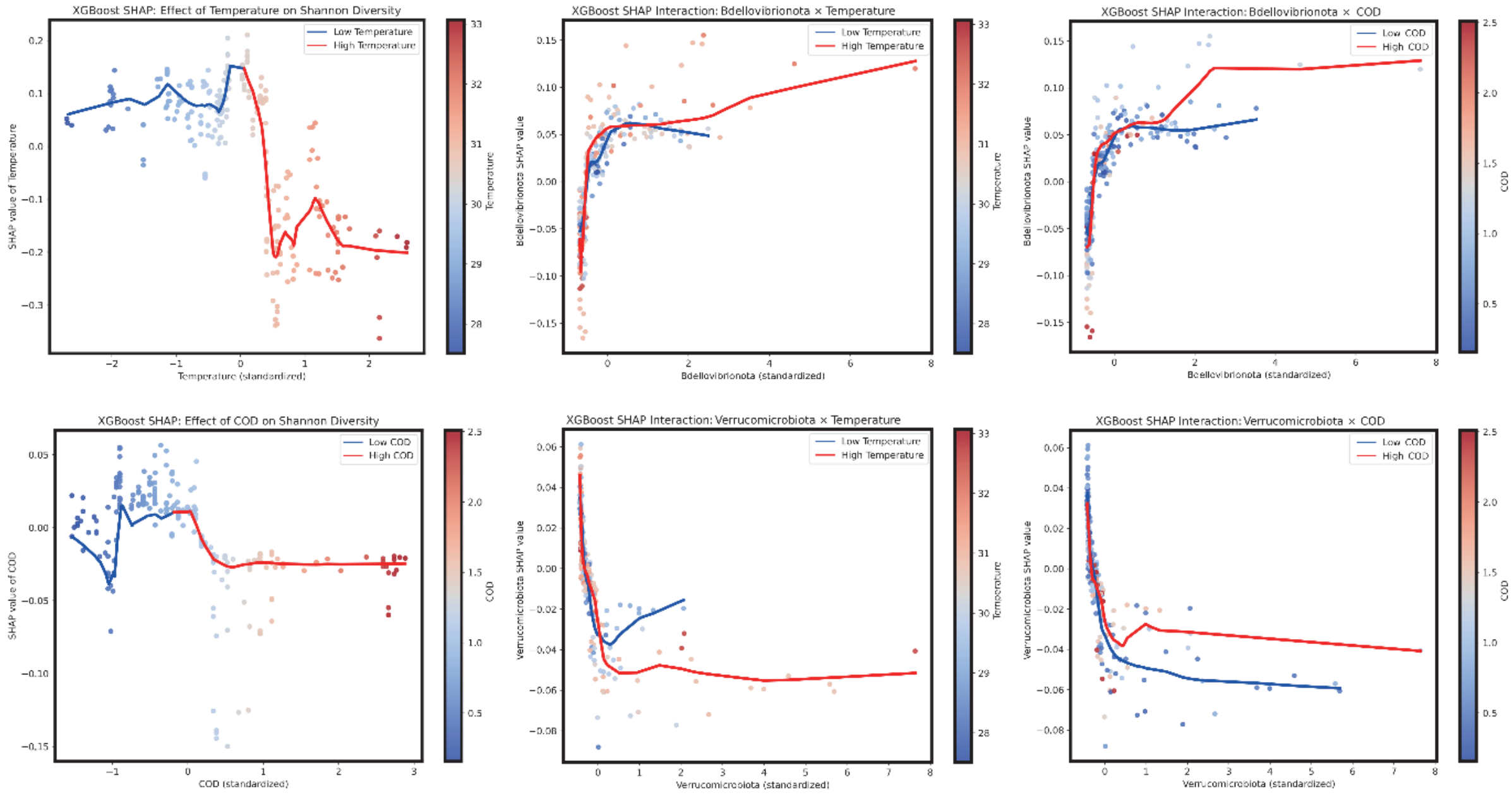

Fig. S10. Interaction effects between single and other factors identified by XGBoost.

Supplementary Figure S11

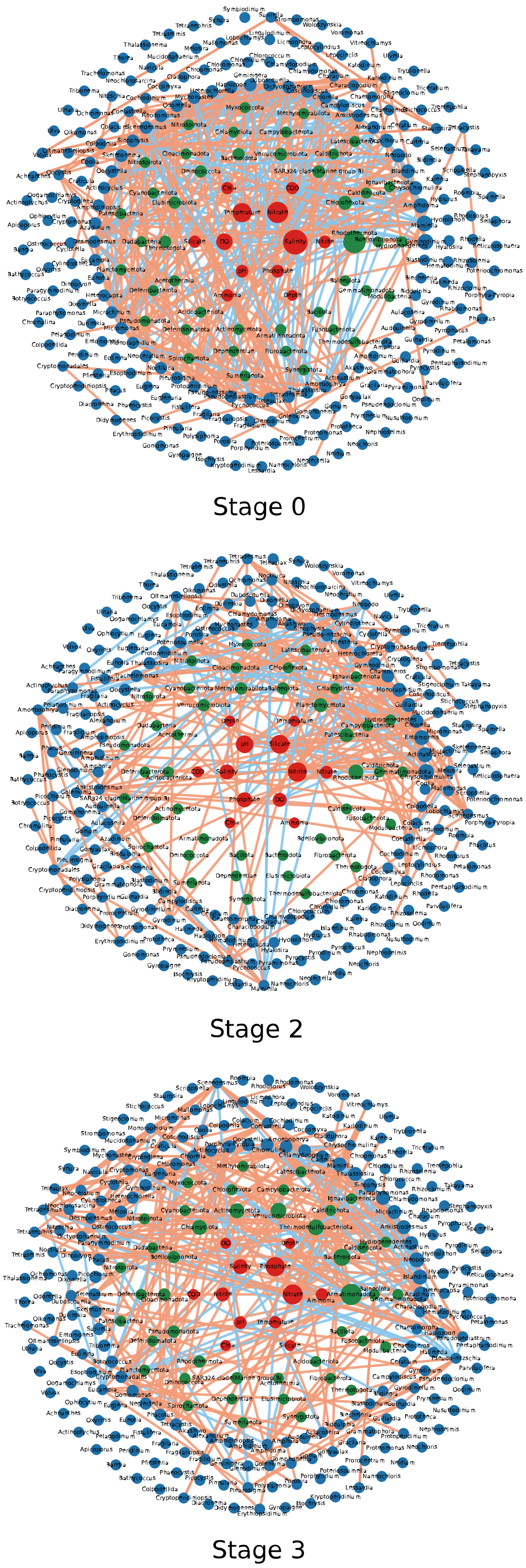

Fig. S11. Panoramic view of bacteria–algae interaction networks across different stages.

Supplementary Tables

Supplementary Table 1. Hyperparameter configurations of models trained on the Prapiroon dataset and the merged dataset.

Model	Prapiroon	Merged dataset
XGBoost	max_depth = 5; eta = 0.1; gamma = 0; colsample_bytree = 0.8; min_child_weight = 1; subsample = 0.6; num_boost_round = 600; objective = reg:squarederror	max_depth = 5; eta = 0.1; num_boost_round = 600; objective = reg:squarederror
Random Forest	n_estimators = 800; max_features = 12; min_samples_leaf = 1; max_leaf_nodes = 60	n_estimators = 600; max_features = 5
SVM	C = 10; gamma = 0.05	C = 1; gamma = 0.05
Elastic Net	alpha = 0.0281; l1_ratio = 0.65; max_iter = 10000	alpha = 0.05; l1_ratio = 0.3; max_iter = 10000
DNN	Layers: 128–64–32–1; Dropout = 0.3; ReLU; Adam (lr = 0.001); Epoch = 300; Batch = 8; Early stopping (patience = 20)	Layers: 128–64–32–1; Dropout = 0.3; ReLU; Adam (lr = 0.001); Epoch = 300 Batch = 8; Early stopping (patience = 20)

Supplementary Table 2. Mean relative abundance of the top five bacterial phyla.

Phylum	Prapiroon	Wipha
Pseudomonadota	41.57	52.92
Actinomycetota	18.33	17.46
Bacteroidota	17.97	10.18
Cyanobacteriota	16.31	16.78
Verrucomicrobiota	2.81	0.37
Bacillota	0.79	0.84

Supplementary Table 3. Mean relative abundance of algal phyla.

Phylum	Prapiroon	Wipha
Bacillariophyta	54.13	32.25
Chlorophyta	24.69	56.94
Pyrrophyta	18.34	8.83
Chrysophyta	1.09	0.81
Cryptophyta	1.13	0.85
Euglenophyta	0.57	0.29
Rhodophyta	0.04	0.017
Xanthophyta	0.01	0.013

Supplementary Table 4. Comparative Path Lengths of Bacterial and Algal Community Responses Between Prapiroon and Wipha.

Type	Typhoon	Total path length
Bacteria	Prapiroon	7.09
	Wipha	2.975
Algae	Prapiroon	8.222
	Wipha	3.25

Supplementary Table 5. Median values of environmental parameters during the two typhoons.

Feature	Typhoon	Stage 0	Stage 1	Stage 2	Stage 3
Temperature	Prapiroon	30.46	30.12	29.69	30.83
	Wipha	31.64	29.17	30.76	29.65
Salinity	Prapiroon	23.06	20.61	16.06	23.88
	Wipha	23.44	22.8	23.42	24.08
pH	Prapiroon	7.82	7.54	7.35	7.75
	Wipha	7.3	7.31	7.32	7.31
Depth	Wipha	1.4	2.1	2.55	2.65
	Prapiroon	2.15	2.15	2.4	2.55
DO	Prapiroon	5.78	4.24	4.26	5.31
	Wipha	4.08	4.72	4.34	4.62
COD	Prapiroon	1.05	0.91	0.91	0.5
	Wipha	1.11	1.1	0.8	0.69
Chl *a*	Prapiroon	1.95	2.4	4.29	5.8
	Wipha	7.9	3.05	3.6	4.36
Nitrate	Prapiroon	0.02	0.03	0.04	0.04
	Wipha	0.86	1.1	0.59	0.6
Phosphate	Prapiroon	0.03	0.05	0.05	0.03
	Wipha	0.1	0.1	0.16	0.14
Silicate	Prapiroon	1.04	1.83	2.85	0.98
	Wipha	1.07	2.37	1.7	0.86
Nitrite	Prapiroon	0.02	0.04	0.12	0.08
	Wipha	0.07	0.05	0.06	0.03
Ammonia	Prapiroon	0.2	0.34	0.36	0.11
	Wipha	0.12	0.49	0.33	0.21

Supplementary Table 6. Confusion matrix of the four-stage classification by the Random Forest model.

True Stage[a]	Pred 0	Pred 1	Pred 2	Pred3
Stage 0	9	4	1	4
Stage 1	1	11	2	4
Stage 2	2	3	9	4
Stage 3	2	2	1	13

[a]Rows represent the true stages and columns represent the predicted stages.

Supplementary Table 7. Classification performance metrics (precision, recall, F1-score, and support) for each stage identified by the Random Forest model.

Stage	Precision	Recall	F1-score	Support
Stage 0	0.64	0.5	0.56	18
Stage 1	0.55	0.61	0.58	18
Stage 2	0.69	0.5	0.58	18
Stage 3	0.52	0.72	0.61	18

Supplementary Table 8. Confusion matrix of the four-stage classification by the XGBoost model.

True Stage[a]	Pred 0	Pred 1	Pred 2	Pred3
Stage 0	9	5	0	4
Stage 1	0	11	3	4
Stage 2	6	4	7	1
Stage 3	3	2	1	12

[a]Rows represent the true stages and columns represent the predicted stages.

Supplementary Table 9. Classification performance metrics (precision, recall, F1-score, and support) for each stage identified by the XGBoost model.

Stage	Precision	Recall	F1-score	Support
Stage 0	0.5	0.5	0.5	18
Stage 1	0.5	0.611	0.55	18
Stage 2	0.64	0.39	0.48	18
Stage 3	0.57	0.67	0.62	18

Supplementary Table 10. Summary of network topology metrics across four stages.

Stage	Nodes	Edges	AvgClustering	LenAver	RadAver
0	100	371	0.488	3.056	7.42
1	88	367	0.42	2.849	8.341
2	92	215	0.396	3.791	4.674
3	105	303	0.45	3.536	5.771

Supplementary Table 11. Edge fluctuations of intra- and inter-group interactions across four stages.

Type	Stage 0	Stage 1	Stage 2	Stage 3	Mean	SD	CV (%)
A-A	358	358	203	290	302.3	73.5	24.3
B-B	28	22	15	48	28.3	14.2	50.3
E-E	69	76	46	36	56.75	18.85	33.2
A-B	30	25	11	50	29	16.14	55.7
A-E	39	35	18	26	29.5	9.4	31.9
B-E	11	12	12	13	12	0.816	6.8
